# Exploring the perspectives of healthcare professionals in delivering optimal oncology medication education

**DOI:** 10.1371/journal.pone.0228571

**Published:** 2020-02-12

**Authors:** Allison Lively, Laura V. Minard, Samantha Scott, Heidi Deal, Tessa Lambourne, Jenn Giffin

**Affiliations:** 1 Department of Pharmacy, Nova Scotia Health Authority (Central Zone), QEII Health Sciences Centre, Halifax, NS, Canada; 2 College of Pharmacy, Dalhousie University, Halifax, NS, Canada; 3 Department of Pharmacy, Nova Scotia Health Authority (Northern Zone), Aberdeen Regional Hospital, New Glasgow, NS, Canada; 4 Nova Scotia Health Authority (Central Zone), QEII Health Sciences Centre, Halifax, NS, Canada; Newcastle University, UNITED KINGDOM

## Abstract

**Background:**

To optimize patient education, it is important to understand what healthcare professionals perceive to be ideal oncology medication education for patients to receive, and what they feel is their role and the role of others in its delivery. Education provided to patients is an important component of chemotherapy as it has been shown to benefit and positively impact patients who receive it. Educational interventions are often provided by multidisciplinary teams with the goal of improving patient care. However, few studies have explored the roles of healthcare professionals in delivering oncology medication education.

**Objective:**

To explore the perspectives of healthcare professionals working in medical, gynaecological or hematological oncology to identify what they perceive to be optimal oncology medication education for patients.

**Methods:**

Healthcare professionals (physicians, nurses and pharmacists) working in medical, gynaecological or hematological oncology at the Nova Scotia Health Authority, Central Zone were invited to participate in one-on-one, semi-structured interviews which were audio-recorded, transcribed and analyzed using thematic analysis.

**Findings:**

Fifteen interviews, including five physicians, four nurses and six pharmacists were conducted from February to April 2018. Four major themes were identified: *Delivery of oncology medication education*, *Facilitating the patient learning process*, *Multidisciplinary Approach and Understanding barriers to the healthcare professional in providing education*.

**Conclusion:**

The identified themes uncovered novel ideas about how healthcare professionals felt oncology medication education could ideally be delivered to patients, and supported findings in the literature. Although participants discussed barriers to their ability to deliver optimal education, they also identified ways in which they can facilitate patient learning, for example, through the reinforcement of education. Participants recognized the importance of increasing collaboration and communication with the multidisciplinary team. This research will inform the design of any new models for oncology medication education at the Nova Scotia Health Authority, Central Zone and potentially other sites.

## Introduction

Educating patients with cancer about their oncology medications is recognized as an important component of therapy as it has been shown to benefit and positively impact patients [1,2]. Patients who receive education feel more equipped to care for themselves, have improved adverse effect monitoring and better treatment adherence [1,2]. Importantly, patients who feel their educational needs have been met by the healthcare team, in general, report a better health-related quality of life and less anxiety and depression about their treatment [2]. However, patients with cancer often report that their educational needs have not been met [3–9].

Oncology medication education can be complex as it may require education on the medications being used to treat the patient’s cancer, medications used to prevent and treat adverse effects related to treatment (e.g. antiemetics, antihistamines, corticosteroids), and medications to treat other aspects of the disease, such as pain. Many of these medications are high-risk and require close monitoring by both the patient and the health care team. Oncology medication education may also include counselling on potential drug interactions that are present between the patient’s oncology medications and other medications they may take at home for comorbid conditions.

The provision of oncology medication education to patients is done by multidisciplinary teams consisting of physicians, nurses, pharmacists, and other healthcare professionals (HCPs). Generally, these HCPs choose what information they will present to the patient to increase adherence and adverse effect management [10,11]. Although the goal of this approach is to improve patient care, discrepancies exist in what information HCPs prioritize compared to the priorities of the patients they treat [5,12–14]. Additionally, little research has explored HCP perspectives in the provision of oncology medication education to discover what they feel is important for oncology patients to know about their oncology medications and what is the role of each HCP in the provision of this education [15].

Educational interventions directed towards oncology patients have focused on improving adherence; however, informational gaps and barriers exist to these approaches [10,11,16,17]. As the educational needs of oncology patients can change over time, patients with unmet needs may be confused over the administration of their medications, have decreased awareness of potential adverse effects and decreased adherence [5,16,18]. Also, many patient factors such as literacy level, psychological adaptation, and the approach of the HCP play a role in their ability to learn and accept education around chemotherapy [5,19].

Pharmacists can have an important role as members of the multidisciplinary team in the provision of oncology medication education. Numerous studies have identified the role of the oncology pharmacist in providing patient education, reducing errors, decreasing the risk of drug interactions and helping patients manage adverse effects [20–23]. In oncology practice in Canada, the role of the pharmacist is stated in The Standards of Practice for Oncology Pharmacy in Canada [24]. These standards recommend that pharmacists provide education to patients on drug adverse effects and management, drug interactions, storage, missing dose information, route, dose and duration of therapy.

At the Nova Scotia Health Authority, Central Zone (NSHA), pharmacist duties include a variety of clinical checks to ensure that intravenous chemotherapy orders are safe and appropriate, as well as the provision of patient education regarding treatment regimens, adverse effects, and drug interactions. However, due to an increased volume of patients receiving chemotherapy, patients often have only brief contact with a pharmacist or they may not be seen by a pharmacist at all. As pharmacists in oncology should be providing education to all patients [24], a new model for patient education needs to be implemented.

Prior to optimizing patient education services at NSHA, multidisciplinary team perspectives need to be investigated. Therefore, this qualitative research study will explore the perspectives of physicians, nurses and pharmacists working in oncology at NSHA regarding what they believe to be ideal oncology medication education for patients, including the timing of this education and what role they believe each team member should have in providing this information to patients.

## Methods

### Study design

A qualitative study design, grounded in the ontological foundation of interpretivism, that reality is socially constructed, was chosen to answer the research objective [25]. Qualitative research methods allow researchers to explore questions of ‘why’ people behave or act the way that they do and can be used to examine institutional and ‘social practices and processes, identify barriers and facilitators to change, and discover the reasons for the success or failure of interventions’ [26]. As our research objective aimed to explore the perspectives of HCPs regarding what they felt was ideal medication education for oncology patients a qualitative approach was taken to explore the thoughts and opinions of the participants [26]. With this approach in mind purposeful sampling was used in this study. Healthcare professionals in oncology (physicians, nurses and pharmacists) were interviewed using semi-structured one-on-one interviews as these interviews maximize the opportunity for a thick description of the data. (S1 Appendix). This interviewing strategy was chosen to allow participants to freely describe their experiences and opinions using open-ended questions. Another advantage of using this type of interviewing is that it allowed for the interview to be scheduled in advance at a designated time and location [27,28]. Ethics approval was granted by the NSHA Research Ethics Board (file # 1023043).

### Participants

Physicians, nurses and pharmacists working in direct patient care in medical, gynaecological or hematological oncology at NSHA were invited to participate in the study. Healthcare professionals who expressed interest in participating in the study were provided with the study information and a consent form. Chemotherapy was broadly defined to include treatment with immunotherapy, targeted, or cytotoxic agents [29].

### Semi-structured interview moderation

Interviews were performed by the principal investigator (AL) and lasted 15–50 minutes. They consisted of semi-structured, open-ended questions (S1 Appendix). Prior to each interview the principal investigator conducted an overview of the topic, allowed an opportunity for questions and reviewed informed consent. No demographic data was collected other than the ‘profession’ (physician, nurse or pharmacist) and ‘discipline’ (medical, gynaecology or hematology oncology) of each participant. Confidentiality was discussed with each participant and participants could agree or disagree to the use of their anonymous quotations in the presentation and publication of results. In addition, the principal investigator signed a pledge of confidentiality. Only the principal investigator had access to the data and pledged not to disclose any participant identifying information.

### Data collection and analysis

Fifteen interviews were conducted between February and April of 2018. These included five physicians, four nurses and six pharmacists. The interviews were audio-recorded and transcribed by the principal investigator. NVivo11 software was used to assist with data analysis [30]. Field notes were taken during and immediately after each interview by the principal investigator to describe the interview setting and to note any additional observations.

Thematic analysis was used to analyze the data [28]. This involved coding transcripts line by line and identifying persistent words and phrases. Codes that were related were grouped together and placed into categories, and themes emerged from the data [28]. Categories and themes that were identified throughout the interview transcripts were constantly compared through multiple cycles of reading and re-reading the transcripts in an iterative process [28]. The interpretation of the data and theme identification was performed with the research question in mind [28]. Thematic analysis was conducted by the principal investigator using the participant interview transcripts. Codes, categories and themes were reviewed by members of the research team throughout thematic analysis. Interviews were completed until no new codes were emerging from the interview transcripts and theoretical data had been achieved [31].

### Rigour and trustworthiness

Several measures were taken to ensure trustworthiness of the data. This included the principal investigator keeping a self-reflective journal during the data collection process to facilitate reflexivity [32]. This approach makes transparent any personal assumptions or biases that may emerge during data analysis [28,32]. Additionally, these notes facilitated the maintenance of an audit trail, and documentation of code changes and decisions around category and theme development. These journals were referred to throughout the data analysis process. An audit trail was maintained by the principal investigator with information on the development of codes, categories and themes. Peer debriefing sessions with research team members (LVM, HD) were also held to further explore the data and challenge data interpretation.

## Results

Fifteen interviews were conducted including five physicians, four nurses and six pharmacists. Four major themes, 15 categories and 63 codes were identified (S2 Appendix). A broad overview of the coding for each HCP group (physician, nurses and pharmacists) is shown in S3 Appendix. When codes for each type of HCP were compared, the proportion of total codes that appeared under each profession was between 71% and 83%. The four major themes were titled: *Delivery of oncology medication education*, *Facilitating the patient learning process*, *Multidisciplinary approach and Understanding barriers to the healthcare professional in providing education*.

### Delivery of oncology medication education

Healthcare professionals had many ideas about how education could be ideally delivered to patients. This theme contained the categories prioritization of chemotherapy drug information, modes of education delivery, presentation of information and timing of education.

#### Presentation of information

“I like simplicity–I like all one stop shopping…one piece of paper [or] two at the most that says it all in less paper” (Pharmacist 3). Presenting the patient with written information was emphasized to be important by all HCPs, but ideally this should be provided in a condensed format. Participants identified patient education needs to be comprehensible. This included presenting drug information using patient-centred language and written material at a grade three or four reading level. “You know, I try not to use big words. Well I mirror actually; I mirror what they bring to me” (Nurse 3). It was identified that patient material is mainly available in English and this can be challenging when speaking to patients in whom English is not their first language. Participants identified that HCPs needed to be transparent when presenting information to patients. They felt the presentation of information should include quantitative values, so that patients are able to understand the risks and benefits of therapy.

#### Prioritization of chemotherapy drug information

All participants emphasized the importance of providing patients with information about how to “troubleshoot at home” to manage adverse effects. This included providing patient education on symptoms related to adverse effects to prevent toxicities related to therapy. When discussing adverse effect information, HCPs prioritized the drug information delivered to patients by discussing “the common and the serious [adverse effects]”. It was identified by physicians that long-term adverse effects of chemotherapy, although discussed initially, are not readdressed again with the patient when they finish treatment: “We don’t do this at all, … where you sit down and you say, ‘so you finished your chemotherapy and these are some of the potential long term [adverse effects]’” (Physician 5). Pharmacists and nurses felt it was important for patients to have a general understanding about how chemotherapy works to kill or target cancer cells and this was mentioned as a component of their teaching that was done with the patient.

#### Modes of education delivery

Participants felt it to be beneficial for patients to have educational material available in a variety of forms. To accommodate different types of learners, providing patients with online resources and videos was felt to be valuable. The importance of providing additional visual information such as medication calendars or showing patients the names of their medication and images of their medications for referral, were described as an essential part of the written and verbal information provided. Physicians discussed novel modes of education delivery that may engage the patient including the use of new technologies (e.g. internet, video) and applications. A participant explained that people “have apps that extract how many steps they’ve taken, you should drink this much water. If we had something like that for chemotherapy and the scheduling for supportive meds and that type of thing that might be helpful too” (Physician 3).

#### Timing of education

Although there was not a clear consensus on this, participants felt that patients needed to have some education provided prior to the first cycle or “in advance of their treatment”. It was felt that patients needed to receive some information and education on the medications they would receive at the time they were being offered treatment. As Pharmacist 5 explained, “[Patients] need that baseline education so that they know–okay, they’re asking ‘do I want to get this chemotherapy…what does that mean?’”. Participants felt that education needed to be repeated for patients. As Physician 2 said, “I think you have to deliver [education] like we are, multiple times. I don’t think you can deliver it once because they don’t hear it.” However, it was also noted that the healthcare team needed to be conscious of the patient’s time and not expect them to return to hospital for more appointments. Nurses recognized that education was often provided to patients at the same time as the treatment was being given: “we teach as we do” (Nurse 1). Education during drug administration was felt to be important from a safety perspective, but was done also as a consequence of limited time.

### Facilitating the patient learning process

Many HCPs generated ideas about how they could assist patients in learning the information they receive. This theme included the categories reinforcement of education, individualized education, understanding patient needs and reassurance.

#### Reinforcement of education

“How many times I’ve heard [patients] say they would sooner hear [information] too many times than not enough. Too many times versus not enough is so much better.” (Nurse 2).

Reinforcing and introducing education by “filling in gaps” or “details” was described by many HCPs. This included asking questions about how medications were taken at home and assessing patient adherence. “A lot of times when [patients] say ‘I have this nausea’–‘Well did you take your anti-nausea pills?’–‘No’”. (Physician 2). To further engage patients, patient-centred teaching sessions were also proposed. “You almost maybe think that if [patients] had a session [where you] tell them—how are you going to take your anti-nausea pills again? or what are you going to do if you get a fever? just so that they—they really absorb the information” (Physician 4). Many HCPs emphasized that education needs “redundancy” and “never stops” for patients.

#### Reassurance

**“**Sometimes [patients] are overwhelmed and sometimes they can’t hear anything, so we do encourage them to [bring] someone with them” (Physician 2). Participants identified that patients need adequate support during education sessions. Family member support was encouraged by HCPs as this support person could help understand the information discussed. Providing the patient with contact numbers so they could reach the clinic and “call with problems” was described as another way to provide reassurance. One physician participant also allowed patients the option of bringing “little recording devices” to appointments. Group education sessions were felt to be beneficial for patients to provide reassurance; patients with similar cancers could be grouped together in these sessions so they could exchange information and support each other.

#### Individualized education

“I don’t know if there is kind of a one fit for everyone” in providing education (Pharmacist 4). Participants emphasized that education is personal and felt it was important to provide an approach that was “tailored to the patient”. Group education sessions were criticized as being very general and it was felt these could be “fine-tuned for a patient” (Physician 3). Healthcare professionals felt it was beneficial to have education provided as a one-on-one session as this allowed an opportunity to provide patient-centred education. However, costs associated with the provision of one-on-one education were felt to be a barrier to its implementation as this would require more staffing resources to provide individualized education to all patients.

#### Understanding patient needs

“Any time you provide information that’s near the diagnosis it’s going to be overwhelming and needs repeating for patients” (Pharmacist 6). Healthcare professionals identified that patients need “time to digest” after the diagnosis. They acknowledged that the patient’s ability to absorb information needs to be assessed when providing education as “it depends on the patient how much depth you can go in” (Pharmacist 5). Participants also thought that developing a relationship with the patient was important for patients to feel comfortable. Physicians acknowledged that it was important to establish patient expectations at the time of diagnosis as this was essential to recommending chemotherapy for the patient.

### Multidisciplinary approach

The use of multidisciplinary teams in the provision of oncology medication education was emphasized as being important for patient care. This theme included the categories collaboration among healthcare professionals, communication among healthcare professionals and differences in healthcare professional roles.

#### Collaboration among healthcare professionals

“I mean ideally it would be nice if there was more collaboration than there currently is. We kind of right now, we all kind of work in our own little silo and we all have different information to give [the patients]” (Pharmacist 4). Ideas for team collaboration involved including pharmacists in chemotherapy clinics as primary educators. Pharmacists felt strongly about being integrated with physicians and nurses in the clinic: “if there was a pharmacist available, [in a] multidisciplinary clinic, then they would have a role, maybe a specialized role, with certain medications” (Pharmacist 2). One physician identified that the use of pharmacists in multidisciplinary clinics could also delegate some of the education done in the consenting process. Each HCP was identified as having a different focus and emphasizing different teaching components which was felt to be beneficial for patients in understanding information. This also highlighted the importance of each HCP in the delivery of oncology medication education.

#### Communication among healthcare professionals

Participants identified a lack of knowledge about what information had previously been provided to the patient by other HCPs. Participants felt this communication gap could be improved with documentation. Written information about adverse effects and drug reactions that patients experienced was felt to be valuable to have documented in the patient file. Participants also identified that documenting education that was provided to patients about their chemotherapy was important. Nurse 2 acknowledged concern about fertility discussions with young patients and chemotherapy: “there’s no way we’re going to start opening up the can of worms of fertility issues the day they are starting treatment”. In addition, sharing educational resources with community HCPs was identified as important: “family doctors should know about this [chemotherapy adverse effect information] because they’re often the first people to hear about toxicities” (Physician 3). Participants also thought community HCPs could reinforce supportive medication teaching; however, it was identified that they may not have the same knowledge or comfort.

#### Differences in healthcare professional roles

Healthcare professionals identified different roles in the delivery of oncology medication education. All participants identified that nurses provide the bulk of chemotherapy education. This education focused on “side effect management” and “cytotoxic precautions” related to chemotherapy. Nurses and pharmacists associated planning, such as scheduling and arranging community supports as part of the nurse’s role. Nurses were also identified to play a significant role “supporting” and “encouraging” patients emotionally as they go through treatment.

Pharmacists were felt to provide “focused” education to patients about their chemotherapy medication in addition to other therapies for chronic diseases. This involved identifying drug interactions or other drug related problems a patient may have while on treatment (e.g., patients at risk of hyperglycemia while on steroids). “They are great any time you have questions about the [chemotherapies] themselves—how the drugs could potentially interact, and they have been great sitting down with patients and going through their medications and making sure there are no [drug] interactions and making specific [recommendations] about the drugs themselves” (Physician 5). They were also identified as having a role in adjusting home medications and suggesting alternatives to therapy with patient drug allergies or intolerances. Pharmacists were acknowledged as being the main educators for patients taking complimentary/natural health products.

Education that physicians provided to patients about their chemotherapy medications included introducing the treatment options, identifying treatment goals and informing patients about the risks. Much of the physician’s time was felt to be spent providing information to the patient on their cancer diagnosis and outcomes.

### Understanding barriers to the healthcare professional in providing education

Healthcare professionals identified barriers they encounter when providing oncology medication education to patients. This theme contained the categories lack of knowledge of other healthcare professionals, current resources, understanding healthcare professional needs and avoiding misleading information.

#### Lack of knowledge of other healthcare professionals

“So yeah I haven’t thought too much in depth who does education because I know what I want to do as long as someone is doing the other things” (Physician 3). Words like “I assume” were used as HCPs described what information was being provided by others. Some participants were not sure how other team members could be integrated into the delivery of oncology medication education. Physician 4 acknowledged uncertainty in how pharmacists could help optimize oncology medication education based on their current interaction, “I don’t know how the pharmacist would intervene [because] I’ve never worked with one in a long time”.

#### Current resources

A lack of staffing resources was identified as a barrier to providing patient-centred education. Pharmacists were noted to spend the majority of their time verifying chemotherapy orders resulting in limited time for patient education in the outpatient setting. “Do [pharmacists] have time to come out of the pharmacy room—the pharmacy office and ask [patients] personally?—No, because if they do that then the other patients are going to be delayed” (Nurse 1). The treatment area where education was often delivered by pharmacists and nurses was described as hectic and lacking privacy which was not ideal for teaching. These HCPs thought this environment would be difficult for patients to discuss private and personal health concerns. Nurse 4 stated, “I mean in an ideal world, if we’re not busy and myself, as the nurse, has time to sit and go over everything and I have [the patient’s] undivided attention—things aren’t beeping off and I am not getting up and going and doing things and coming back to start over or pick up where I left off”. This setting for education delivery was felt to be distracting and not conducive for learning.

#### Understanding healthcare professional needs

In response to quickly evolving chemotherapy treatment regimens, HCPs identified a desire to stay up to date with in-services or “education refreshers” to improve patient care. “What used to be the ‘gold standard’ for treating certain diseases has evolved over the years and now treatment can consist of second line therapy or things you have never heard of” (Nurse 3). Participants also identified a desire to work to their full scope of practice with the introduction of specialized practitioners in the clinic to provide education and perform assessments.

#### Avoiding misleading information

Healthcare professionals identified a number of ways patients can be provided with misleading information during chemotherapy education. Group teaching sessions could potentially create confusion as patients with different diagnoses and treatments are grouped together. “I do think they need to have differences between intravenous [chemotherapy] and the immunotherapies because if you don’t have that divide they’re not the same” (Physician 5). A lack of educational resources available for patients receiving immunotherapy or oral chemotherapy was also identified. To improve this gap in care, HCPs thought efforts should be in place to better support these patients. Available educational resources were also identified to be a potential source of confusion if not reviewed first. As Pharmacist 4 explained, “Sometimes our protocols don’t match well with the educational resources we have available to us through the [British Columbia] Cancer Agency, so if that’s the case I don’t give it to the patients because I feel that’s just confusing them more”.

## Discussion

Using one-on-one interviews, the perspectives of HCPs working in oncology at NSHA were explored to identify what they perceive to be ideal oncology medication education for patients to receive. Some of the content has been supported in the literature; however, new information was also uncovered. Importantly, two previous studies have explored the perspectives of oncology patients at NSHA regarding optimal medication education delivery [3,5]. By studying both patients and HCPs from the same site, we have identified several key areas where patient opinions aligned with those of the HCPs, which will allow us to strategically design a new model of education to enhance patient care at NSHA.

### Delivery of oncology medication education

It was emphasized that patients should receive written and verbal education on chemotherapy focused on the common and serious adverse effects. Written information about chemotherapy medications and medication schedules were identified as important by HCPs as this provides patients with something to refer to at home, a finding which has been identified from previous studies at our site as well as elsewhere in the literature [3,5,33].

Physicians felt that the use of new technology and applications for education delivery should be explored in the future to engage patients and this could be an area of focus in educational development. Research has also found that using an intervention such as a web-based tool to self-report symptoms had a survival benefit for patients receiving oncology treatment [34]. Other cancer centres have used video and applications to increase access to educational resources for caregivers, and patients at NSHA have also expressed a desire to have access to reliable internet sources to increase information access [3,5,35].

All participants acknowledged that patients needed to receive some education initially about their oncology medications as this was crucial to making an informed decision about treatment. To better utilize patient time, follow-up phone calls could be offered to reduce the number of trips patients are required to make to the hospital. Forty-three percent of patients at NSHA have reported that they would be interested in receiving follow-up from a hospital pharmacist [3].

### Facilitating the patient learning process

Healthcare professionals expressed that chemotherapy education is personal and should be tailored to the patient, a finding also expressed by oncology patients [5,35,36]. The use of patient-centred education sessions was also felt to be beneficial in engaging patients. Patient-centred discussions allow the patient to centre the conversation around their concerns and HCPs can assess comprehension by asking the patient questions. Patients have also expressed a desire to have education provided to them using a teach-back method, which has been recommended to increase patient satisfaction [5,37]. Healthcare professionals felt education was continuous and that offering more frequent patient-centred education options could help increase patient information retention and allow additional opportunities for questions, another concept emphasized by patients [3,5]. As patients are presented with an overwhelming amount of information at the time of diagnosis, these approaches could improve information absorption and allow time to formulate questions as patient coping mechanisms are often initially impacted at the time of diagnosis [33]. Additional ways to support the patient were identified such as family member presence and group education for patient support. These were felt to be important for patient comfort and reassurance, which has also been supported in the literature [5,35].

### Multidisciplinary approach

Healthcare professionals emphasized the need to work together in the delivery of oncology medication education since all professions agreed that everyone has a role. They identified that the incorporation of pharmacists into multidisciplinary clinics would be useful in providing patient education and identifying drug related problems. The use of specialized oncology pharmacists has also played a role in chemotherapy cost savings and improving the quality of patient care at other sites [38,39]. The need for improvements in communication was stressed by participants and multidisciplinary teams could focus on approaches to improve documentation and communication among HCPs. To increase documentation among HCPs some participants thought oncology medication educational checklists could be developed. These checklists could be incorporated into the patient chart to help increase awareness among team members of what information has been previously discussed with the patient. Improvements in oncology documentation have been tried at other sites using the electronic health record which may be a resource worth exploring to improve documentation [40].

### Understanding barriers to the healthcare professional in providing education

Healthcare professionals acknowledged that they may not fully understand the scope of practice of each HCP working in the team and noted a lack of understanding over what information other team members provided to patients. This is important to recognize when planning to optimize oncology medication education provided by multidisciplinary clinics as the roles of each member of the healthcare team should be established [15]. Barriers identified which hinder education delivery included a lack of time with the patient, staffing resources, hectic work environment and privacy. Many HCPs identified gaps in the delivery of oncology education for patients receiving targeted therapies or oral chemotherapy as currently there are few resources available. Recognition of these educational gaps by both HCPs and patients will provide opportunities for HCPs to design novel educational resources at the NSHA to improve patient safety and care [5].

Participants also recognized the need to review and optimize educational material to ensure that information received by patients is accurate and to avoid the distribution of misleading information. In particular, some material presented to patients at NSHA during group education sessions may not apply to all patients. Additionally, participants highlighted the need to review educational material being handed to patients as most resources are not specific to NSHA and there may be slight variances in some of the recommendations, which can contribute to patient confusion.

#### Strengths and limitations

Strengths of this research include the incorporation of multidisciplinary perspectives using a qualitative design to obtain rich research data which would not have been possible using quantitative methods. Steps were taken to ensure participant confidentiality and promote an open discussion during the interviews (See Rigour and Trustworthiness). Although we believe that theoretical data saturation was achieved with 15 participant interviews, it is unknown whether more codes would have emerged with more interviews [31,41]. Another limitation is that the themes identified in this research relate to the opinions of HCPs working in oncology at NSHA and may not be transferable to other institutions.

#### Implications of findings and recommendations

This research describes the HCP perspective of optimal oncology medication education at NSHA and follows up on previous studies that have examined the patient perspective. Facilitators to education delivery such as verbal communication and written material have previously been supported by patients receiving oncology medication education and this formatting should be maintained in future medication education models [3,5,33]. Additionally, patients being treated at NSHA have expressed a desire to have reliable internet resources available to them [3,5]. Standardization of written information and recommended online resources could help to avoid the distribution of misleading information. As outlined by HCPs, the use of new technology such as applications or web-based resources could also be explored.

Healthcare professionals identified that patients with similar cancers could be grouped together in education sessions. Patients have also identified that access to patient support groups should be a component of education as hearing others’ experiences can help lessen anxiety and confusion [5]. Therefore, future changes in oncology education sessions should focus on trying to group patients with similar diseases and treatments together.

Since the educational needs of patients change over time, and both HCPs and patients have identified the need for continuous education, it may be beneficial to allow patients the opportunity to schedule additional education sessions, such as follow-up phone calls or appointments with pharmacists [3,5]. Offering follow-up phone calls gives patients an opportunity to absorb and formulate questions, provides the patient with privacy for questions that may not feel comfortable asking in a treatment area and also tailors the discussion to the patient. These were all described as barriers to education delivery in the current model [3,5]. Telephone follow-ups could be piloted in any future education models that are developed. Knowledge gained from this research will be translated within our facility and a multidisciplinary approach in the provision of oncology medication education is recommended in which there is an expanded clinical role of the pharmacist.

The results of this research will be used to improve medication education delivery to oncology patients by HCPs at NSHA, and to help guide any new educational resources that are developed to improve patient-centred care and the quality of oncology education provided to patients. This research may also promote multidisciplinary teams providing oncology medication education to patients, and further promote pharmacist clinical services in oncology at NSHA and at other institutions. More research is needed to determine whether these results apply to the broader oncology HCP population.

## Supporting information

S1 Appendix Interview question guide. (DOCX)

S2 Appendix Thematic analysis: Themes categories and codes. (DOCX)

S3 Appendix Coding results for healthcare professionals. (DOCX)
