# Supplementary material for: Exploring the perspectives of healthcare professionals in delivering optimal oncology medication education
Source: PLoS One. 2020 Feb 12;15(2):e0228571. doi: 10.1371/journal.pone.0228571 (PMC7015363; doi:10.1371/journal.pone.0228571)
Supplement: S1 Appendix — (DOCX) [file pone.0228571.s001.docx]

S1 Appendix. Interview Question Guide

Question 1:

What oncology medication education do you provide to patients?

*Prompts:*

*When is that education provided?*

Question 2:

In a collaborative environment with nurses, physicians and pharmacists working together, what do you think the role is for each healthcare professional in providing patient education on oncology medications?

*Prompts:*

*Think about an ideal situation?*

*How could you see the different team members delivering education differently?*

*What could the other professions do that they are not doing now?*
