# Supplementary material for: Exploring the perspectives of healthcare professionals in delivering optimal oncology medication education
Source: PLoS One. 2020 Feb 12;15(2):e0228571. doi: 10.1371/journal.pone.0228571 (PMC7015363; doi:10.1371/journal.pone.0228571)
Supplement: S2 Appendix — (DOCX) [file pone.0228571.s002.docx]

| **Theme** | **Multidisciplinary approach** | | | | |
| --- | --- | --- | --- | --- | --- |
| **Categories** | *Collaboration among healthcare professionals (HCPs)* | *Communication among oncology and community HCPs* | *Distinct roles of the HCP in education delivery* |  |  |
| **Codes** | Collaborative approach  (B1-3,6): 4  (C1): 1 | Interdisciplinary communication  (A1-2,4): 3  (B3): 1 | Role of the pharmacist  Adjustment of patient home medications:  (A2) 1  (B2): 1  (C2): 1  Alternative to medications  (B5): 1  Assessing chemotherapy doses:  (A3): 1  (B3): 1  (C4, D1): 2  Assessing chemotherapy toxicity:  (B3-4): 2  (C3): 1  Chemotherapy medication education:  (A1-3): 3  (B3, B5-6): 3  (C2-4, D1): 4  Complementary medication education: (A1-3): 3  (B5): 1  (C4):1  Consenting for chemotherapy:  (C1): 1  Detailed drug history:  (A2): 1  (C4): 1  Drug coverage: (B5): 1  Drug Interactions with chemotherapy:  (A1-4): 4  (B2): 1  (C2-3, D1): 3  Medication Reconciliation:  (B4): 1  (C4): 1  Pharmacist clinic:  (B1-3,5): 4  (C1, D1): 2  Writing chemotherapy orders:  (C1): 1 |  |  |
|  | Different perspectives of different HCPs in delivering education  (A2): 1  (B2,5,6): 3  (C2, D1): 2 | Involvement of community HCPs  (A4): 1  (B5-6): 2  (C3): 1 | Role of the nurse  Arranging teaching:  (A1-2): 2  (C2): 1  Assessing compliance:  (C4): 1  Collecting patient information:  (B3): 1  Emotional support for the patient:  (A2): 1  (B1): 1  (C2): 1  Filling in gaps: (B2): 1  General chemotherapy medication education:  (A2-4): 4  (B1-2,5-6): 4  (C1,4): 3  Reiteration of teaching:  (C1, D1): 2  Supportive medication teaching:  (A3): 1  (B1-2,4-5): 4  (C2-4, D1): 4  Toxicity assessments: (B3): 1 |  |  |
|  | Teamwork  (A1-4): 4  (B2-3, B5): 3  (C1,D1): 2 |  | Role of the physician  Cancer diagnosis: (A2-4): 3  (B1-2,4): 3  (C1-2,4): 3  Different treatment options:  (A2,4): 2  (B3,5): 2  (C2,4): 2  Goals of chemotherapy: (B2-3): 2  (C1,3-4, D1): 4  Informed consent:  (A1,3-4): 3  (B1,3-4): 3  (C1-4): 4  Introduction of chemotherapy treatment:  (A1): 1  (D1): 1  Patient reassurance:  (C1): 1  Prescriptions for supportive medications: (A3): 1  (B1): 1  Risks of treatment:  (A2): 1  (B1): 1  (C3, D1): 2 |  |  |
|  | Multidisciplinary clinic  (B1-3,6): 4  (C1): 1 |  |  |  |  |
|  |  |  |  |  |  |
| **Theme** | **Delivery of oncology medication education** | | | | |
| **Categories** |  | *Presentation of information* | *Prioritization of chemotherapy drug information for patients* | *Modes of education delivery* |  |
| **Codes** |  | Condensed information  A3: 2  (B1,3,5-6): 7  (C4-5) | Explaining how chemotherapy works (Explaining to a patient how chemotherapy attacks cancer cells and healthy cells)  (A1,4): 5  (B1,6): 3 | New technologies (this may be able to go under Apps, however it was vague and could be interpreted differently)  (C2): 2 |  |
|  |  | Comprehensible information  (A1-3): 7  (B3,5): 3  (C2,4): 2  (D1): 1 | Importance of monitoring (Patient understanding the importance of testing as a part of drug therapy)  (A1): 1  (B3,5): 3 | Apps (this may be able to go under new technologies however it was specific so I identified it differently)  (C3): 1 |  |
|  |  | Transparent information  (B4,5): 2  (C1,2): 3 | Prioritizing side effect information (Organizing side effect information for patients so they understand common and rare side effects)  (A2-4): 8  (B1,3-5): 8  (C1-3): 12  (D1): 1 | Internet  (C3): 1  (D1):1 |  |
|  |  |  | Preventing side effects of chemotherapy (Going through information with patients on how to prevent side effects)  (A1-4): 5  (B1,3-4,6): 5  (C2-3):2  (D1): 1 | Written and verbal information  (B3,6): 3  (C1,3-4): 3 |  |
|  |  |  | Preventing infections (Measures that are taken to prevent infections):  (A1,4): 2 |  |  |
|  |  |  | Alarm symptoms  (A1,3-4): 8  (B1-2,5-6): 6  (C1-2): 2 | Multilingual  (A2): 1  (C2): 4 |  |
|  |  |  | Long term side effects  (C3): 1  (D1):2 | Specific chemotherapy drug written information  (A1,4): 4  (B1,4-5): 3  (C2-4): 4  (D1): 3 |  |
|  |  |  |  | Visual aids  (A1-2,4): 8  (B1,3): 3  (C1,4): 4 |  |
|  |  |  |  | Video  (A3-4): 3  (C3): 1  (D1): 1 |  |
| **Theme** | **Facilitating the patient in the learning process** | | | | |
| **Categories** | *Timing of education* | *Reinforcement of education* | *Individualized education* | *Reassurance* | *Understanding patient needs* |
| **Codes** | Education prior to starting treatment  (A2-4): 3  (B1,3-6): 8  (C1,3): 6 | Filling in gaps  (A3-4): 2  (B1,3,5-6): 11  (C1,4): 4  (D1): 2 | Tailored to the patient  (Includes *Tailored to an older population*)  (A1-4): 19  (B2-5): 7  (C1-4): 15  (D1): 4 | Family member support  (A1-2,4): 6  (B1,5): 2  (C1-2): 3 | Time to digest  (A1-4): 9  (B1-6): 16  (C1-4): 14  (D1): 3 |
|  | Education prior to administering chemotherapy (Nurses felt this was the time they gave education to patients this may have been a consequence of time with the patient)  (A1-3): 7 | Repetition of chemotherapy education  (*Reinforcement of education was changed and put between the repetition of education and also monitoring side effects*)  *Includes: Continuous education throughout treatment*  (A1-4): 8  (B1-3, 5-6): 11  (C1-4): 14  (D1): 5 | Engaging the patient in their care (having the patient interact with their material not just a pile of papers)  (C3): 1 | Group support  (A3): 1  (C1): 1 | Developing a patient relationship  (A2): 1  (B1,5): 2  (C1-2): 3 |
|  |  | Assessing compliance  (A1-2,4): 7  (B4): 1  (C2,4): 3 | Utilization of the patient’s time (*Has to do with tailoring the education to the needs of the patient and not expecting them to come in multiple times for education*)  (A3): 1  (B3): 1  (C1,3-4): 5 | Contact numbers  (A3): 1  (C1-2): 3 | Psychosocial aspects of chemotherapy  (A1-3): 6  (B1): 1 |
|  |  | Monitoring side effects  (A1): 1  (B3,5): 3 | Privacy when providing education  (A2,4): 3  (B3-4): 2 | Recording devices (this was stated specifically in the context of the patient having it for support so they could replay information)  (C2): 1 | Patients get overwhelmed  (A1-3): 14  (B1,4-5): 7  (C2,4): 3  (D1): 1 |
|  |  |  | One on one education  (A2-3): 3  (B1,5): 4  (C3-4): 4 |  | Volume of information  (A1-4): 10  (B1,3-5): 5  (C1-2,4): 6 |
|  |  |  |  |  | Patient expectations  (C1,4): 8 |
| **Theme** | **Understanding barriers to the HCP in providing education** | | | | |
| **Categories** | *Needs of HCP* | *Lack of knowledge of other HCPs* | *Current resources* | *Avoiding misleading information* |  |
| **Codes** | HCP working within their scope of practice  (A3): 1  (B3,6): 2 | Assumptions  (*Include fertility and chemotherapy as this is used as an example as information one HCP professional assumes the MD goes over with the patient*)  (A1,3): 3  (B1): 1  (C3): 1 | Hectic environment  (A1-4): 10  (B1): 1 | Standardization of information  (A4): 1  (B4-5): 2  (C3-4): 1, 5  (D1): 2 |  |
|  | Delegation of workload (one participant placed a lot of value on delegating the workload among the HCP team members)  (C1): 9 | Lack of knowledge of other HCP roles  (this also included “I don’t know what they tell them” which was a code where participants discussed what they thought other HCP were providing to patients – there are similarities here to the information in Assumptions)  (A2,4): 2  (B3-5): 3  (C3-4): 5 | Time constraints  (A1-4): 15  (B3): 3  (C1-2): 6 | HCP checklists  (A1): 3  (C3): 1 |  |
|  | Training of staff  (A3): 4  (B3,5): 2  (C3): 1 |  | Staffing resources  (A1,4): 4  (B1-6): 24  (C1,3-4): 9  (D1): 3 | Accurate information  (B3): 1  (C3): 2  (D1): 2 |  |
|  |  |  | HCP expectations of the patient (this used to be related to patients not studying the information that we gave them. After review this changed to what we as HCP expect patients to do. We want/expect them to review the reading material we provide them with)  (A2): 1  (C1-4): 7 | Conflicting information  (A3): 2  (B3-4) 6  (C3): 3  (D1): 2 |  |
|  |  |  |  | Lack of information on immunotherapy  (B2): 1  (C3): 2  (D1): 5 |  |
|  |  |  |  | Updated resources  (B5): 1  (C2-3): 3 |  |
