# Supplementary material for: Exploring the perspectives of healthcare professionals in delivering optimal oncology medication education
Source: PLoS One. 2020 Feb 12;15(2):e0228571. doi: 10.1371/journal.pone.0228571 (PMC7015363; doi:10.1371/journal.pone.0228571)
Supplement: S3 Appendix — (DOCX) [file pone.0228571.s003.docx]

|  | **Physicians** | **Nurses** | **Pharmacists** | **Total** |
| --- | --- | --- | --- | --- |
| **Number of participants** | 5 | 4 | 6 | 15 |
| **Total number of codes** | 52/63 (83%) | 46/63 (73%) | 45/63 (71%) | 63 |
